# Proposed Standards for Implementing Stepped Care Models in Child and Youth Mental Health Service Systems: Results of a Pan‐Canadian Delphi Study

**DOI:** 10.1111/eip.70057

**Published:** 2025-05-30

**Authors:** S. Mughal, B. Young, A. Churchill, J. Rash, K. Tee, A. Salmon, J. L. Shah

**Affiliations:** ^1^ Department of Psychiatry McGill University Montreal, QC Canada; ^2^ Douglas Mental Health University Institute Montreal, QC Quebec Canada; ^3^ Stepped Care Solutions Mount Pearl, NL Canada; ^4^ Department of Psychology Memorial University of Newfoundland St. John's, NL Canada; ^5^ Foundry Vancouver, BC Canada; ^6^ Centre for Advancing Health Outcomes University of British Columbia Vancouver, BC Canada; ^7^ School of Population & Public Health University of British Columbia Vancouver, BC Canada; ^8^ Department of Epidemiology, Biostatistics and Occupational Health McGill University Montreal, QC Canada

**Keywords:** adolescent, child, Delphi technique, mental health services, standards, stepped care models

## Abstract

**Background:**

Stepped care (SC) is being adopted in many countries as a framework for organising mental health care in diverse contexts. However, there is a lack of consistency in how SC has been defined and operationalised, limiting its effective application in practice. We describe the development of standards for implementing SC models in Canadian child and youth mental health (CYMH) contexts using a consensus‐based approach. These standards are intended to support systems planners in creating cohesive CYMH systems across Canadian settings.

**Methods:**

This study employed learning alliance and Delphi methodologies. A pan‐Canadian multi‐round Delphi process conducted in English and French was used to derive consensus on the inclusion and wording of individual clauses in the standard. Consensus with a threshold of 70% was set to determine the inclusion of individual clauses in the final standard.

**Results:**

Sixty‐eight individuals participated in the Delphi study (with a 76.48% retention rate) representing lived experience, service delivery, policy, and research expertise. Over three rounds, 29 clause items were revised and reduced to a final list of 24 clause items comprising SC implementation standards. Participant feedback indicated a desire for reduced ambiguity, considerations of the limitations of patient autonomy, and the need to clarify roles and responsibilities in system‐wide activities.

**Discussion:**

The results of this Delphi study represent the first multi‐stakeholder, consensus‐driven set of standards for implementing SC in CYMH settings across Canada. With these standards, we aspire to provide a blueprint for mental health systems advocacy and reform toward stronger, more coordinated CYMH systems.

## 1 Introduction

Mental health systems are characterised by high fragmentation, lack of coordination, and an insufficient capacity to provide adequate care to those in need, with resulting poor outcomes (Malla et al. 2021; Iyer et al. 2019). Stepped care (SC) is increasingly being adopted around the world as a framework for organising mental health care in diverse contexts, including in schools (Bailey et al. 2022; Gellatly et al. 2024), on digital platforms (Jagayat et al. 2024), and in diverse community settings (Mediavilla et al. 2023; Wuthrich et al. 2021). These models, which outline the arrangement of services along a continuum of support and intensity, have gained recognition due to their perceived potential to enhance accessibility (Bailey et al. 2022), improve system efficiency (Richards et al. 2012), and ensure that a comprehensive range of interventions is available to meet the varied needs and preferences of entire communities (Cornish 2020). The need for systemic reforms consistent with the goals of SC is particularly pronounced for Canadian children and youth, given that the majority of mental illnesses emerge before the age of 25 (Caspi et al. 2020), and an estimated 41% of youth in Canada do not receive mental health treatments when needed (Gorfinkel et al. 2023).

A key challenge in implementing SC is the lack of consistency in how it has been defined and subsequently operationalised. In explorations of SC in practice (Richards et al. 2012; van Straten et al. 2015), including in child and youth service settings (Berger et al. 2021), substantial variability was observed in nearly every aspect of SC model development and implementation. Self‐identified SC models differed in the number of steps or services offered, the types of services provided (e.g., low vs. high‐intensity interventions, the inclusion of specialist services), available access points and triaging processes, and target populations (e.g., limiting treatment to a single mental illness vs. providing transdiagnostic care across populations). Meta‐analyses have observed SC to be effective for improving patient outcomes (van Straten et al. 2015; Jeitani et al. 2024); however, heterogeneity in SC implementation limits the ability to identify any specific component(s) that consistently contribute to effectiveness (van Straten et al. 2015).

**FIGURE 1:**
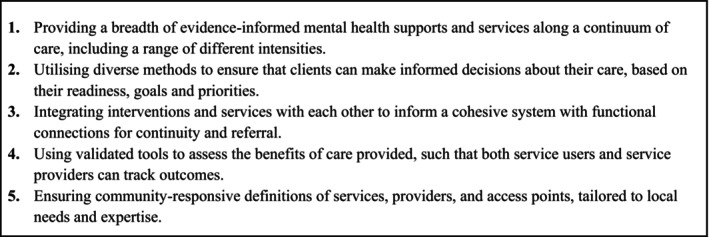
Guiding principles for implementing stepped care in mental health (Mughal et al. 2023).

While no two mental health systems are identical, the lack of consensus on definitions and core components of SC restricts its applicability and creates confusion. It also impedes evidence‐informed implementation and limits the concept's potential to guide critical elements for consistent, positive outcomes. In an effort to align the field and address this research gap, five guiding principles have been articulated as a starting point to assist system planners in applying SC (Figure 1) (Mughal et al. 2023). These principles organise SC implementation around common values and priorities, though they do not offer sufficient, specific direction for implementation. Building on these principles, we now describe the consensus‐based derivation of novel standards for implementing SC models intended to support system planners in creating cohesive child and youth mental health (CYMH) systems in differing Canadian contexts.

This overall project had three objectives: (1) to engage youth and families with lived experience and develop a learning alliance; (2) to translate guiding principles to implementation standards via consensus methods; and (3) to operationalise and apply the resulting standards to three exemplar CYMH service ecosystems (Young et al. 2024). This manuscript focuses on the results of Objective 2.

## 2 Methods

### 2.1 Overview

This study employed learning alliance and Delphi methodologies, leveraging a broad network of interdisciplinary experts to create novel standards. A learning alliance was chosen to guide the development of standards as this method fosters collaboration among diverse stakeholders to build capacity, share best practices and develop collaborative solutions to complex problems (Moreno‐Leguizamon et al. 2015). The learning alliance consisted of participants from across Canada, including English and French‐speaking individuals representing a range of perspectives (Young et al. 2024).

### 2.2 Delphi Participants

The Delphi method enables consensus‐building through iterative rounds of anonymous feedback, allowing the draft to iteratively evolve without judgement within the group and minimising bias from potential power imbalances (e.g., between clinicians and youth with lived experience) (Hsu and Sandford 2007). Delphi participants were a subset of self‐selected learning alliance members who committed to completing all rounds of the Delphi process. To ensure diverse perspectives, participants were selected from across each of Canada's regions (i.e., Atlantic, Central, Prairie, West, and Northern regions) and asked to select their primary and secondary stakeholder affiliations from five groups. Additional purposive sampling was conducted via members of the learning alliance to bolster representation across geographic, demographic and stakeholder groups.

Participants provided electronic informed consent prior to beginning the Delphi study using the Calibrum platform (Calibrum, n.d.). Consent was provided from a parent/guardian on behalf of participants under the age of 18. Participants who denoted a primary affiliation in either Lived Experience category were compensated for their time at a set rate. Onboarding of participants was supported with online learning modules prepared by the research team ahead of Round 1 (Young et al. 2024).

### 2.3 Delphi Procedure

Surveys were conducted digitally using Calibrum, software specifically developed for Delphi surveys (Calibrum, n.d.). To populate the survey, an initial list of 29 clause items was developed by the research team. This list was divided into five categories, one for each of the aforementioned five guiding principles (Figure 1) (Mughal et al. 2023), and was revised through two focus groups with youth and families who were not participating in the Delphi surveys (Young et al. 2024). Each survey consisted of this list of clause items and was provided in English and Canadian French.

In each round, respondents were asked: “To what extent do you agree or disagree with the inclusion of this item in our standard?” They were instructed that standards are, by definition, aspirational and that participants should disregard resource constraints or locally specific feasibility issues when completing the Delphi, as the latter could hinder the development of a broad standard of practice. Respondents were asked to rate their agreement with each draft clause on a 6‐point Likert scale:

1. “Disagree, Strong Objections”
2. “Disagree, Mild Objections”
3. “Neutral, Leaning Disagree”
4. “Neutral, Leaning Agree”
5. “Agree, With Revisions”
6. “Agree, As Written”

The 6‐point scale was selected to remove a neutral option, aiding in Delphi analyses and identifying respondent agreements and disagreements. An open text box was also provided for suggested revisions, comments and questions for each clause. Respondents were also given the opportunity to provide feedback on the full list of clause items at the end of the survey.

### 2.4 Delphi Analysis and Standards Derivation

Consensus was the primary measure for determining whether an individual clause item would “pass” (i.e., be included in the final standard) or was to be removed from consideration. Following common Delphi procedures (Hsu and Sandford 2007; Hasson et al. 2000), a threshold of 70% was required to reach consensus. This threshold was applied within each stakeholder group (i.e., a consensus threshold of 70% agreement rating of 5 or 6 in *each* individual group was required to “pass” a clause). This criterion was set to ensure that all stakeholder perspectives were equally represented in decisions between rounds.

**FIGURE 2:**
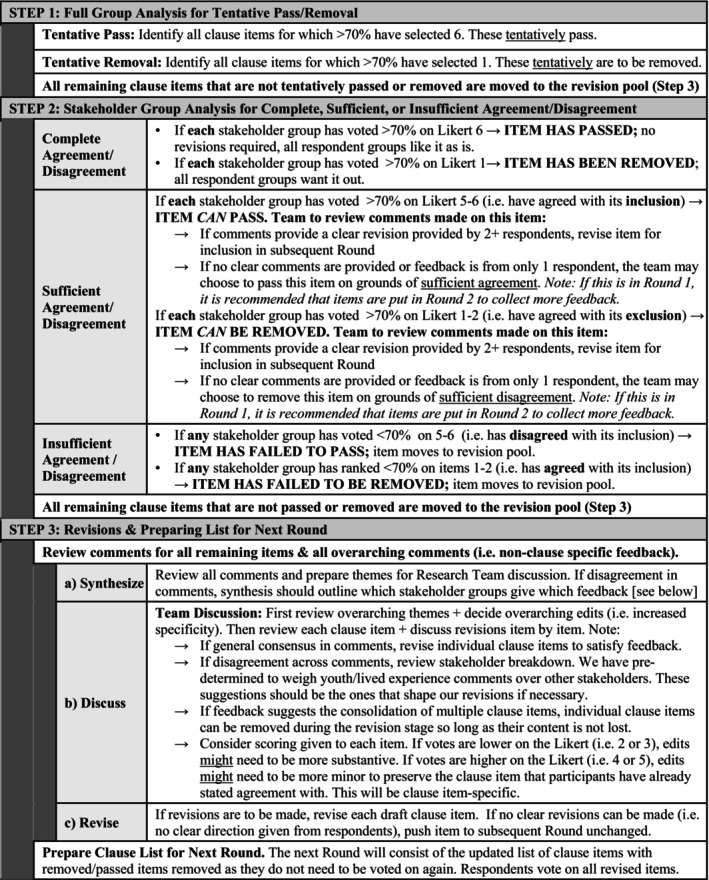
Decision support tool for Delphi responses.

Data analysis for each round included three steps. First, scores for each clause item were reviewed across groups to identify items that were tentatively passed or tentatively removed. Second, individual stakeholder‐group scores for each tentative pass/remove item were reviewed to identify if sufficient agreement across groups was achieved to pass/remove each item. Third, comments were reviewed and categorised for all clause items that were neither passed nor removed to guide revisions. Comments categorised as “Revision” were thematically analysed, and relevant revisions were discussed with the research team. These clause items were then revised and placed in a subsequent Delphi round. In line with our commitment to patient‐oriented research, feedback from lived experience stakeholders was weighted more strongly than that of other stakeholders when revising items in cases where comments were discrepant across groups. This process was repeated over three rounds of Delphi surveys. Additional details on these data analyses and revision procedures are outlined in Figure 2. An example of the individual clause revision process across rounds is outlined in Figure S1.

### 2.5 Ethics

All procedures contributing to this work comply with the ethical standards of the Helsinki Declaration of 1975, as revised in 2013. Procedures were approved by the Centre intégré universitaire de santé et de services sociaux (CIUSSS) de l'Ouest‐de‐l'Île‐de‐Montréal—Mental Health and Neuroscience subcommittee (REB #2024‐958). Informed consent forms, including parental/guardian consent forms, were created with approval from the same research ethics board.

## 3 Results

### 3.1 Delphi Participants

Sixty‐eight participants registered for this study. The most frequent primary stakeholder affiliation was Service Delivery (45.59%), and the least frequent was Policy/Decision‐Making (4.41%). Stakeholder group proportions widened when participants were asked to select all that apply; the most frequently selected group was Lived Experience—Other (77.94%), and the least frequent was Policy/Decision‐Making (20.59%). Attrition was proportional across stakeholder groups, with a final retention rate of 76.48% (Table 1).

**TABLE 1:** Participant stakeholder affiliations across rounds 1–3.

Delphi round	Round 1		Round 2		Round 3	
	*N* = 68		*N* = 55		*N* = 52	
Primary Stakeholder Group Affiliation (note: participants could only select one)						
Lived Experience—Self	13	19.12%	11	20.0%	11	21.1%
Lived Experience—Other	10	14.70%	12	21.8%	9	17.3%
Service Delivery^a^	31	45.59%	20	36.4%	20	38.5%
Policy/Decision Maker	3	4.41%	3	5.5%	2	3.8%
Researcher	11	16.18%	9	16.4%	9	17.3%
Unknown	—	—	—	—	1	1.9%
Additional Stakeholder Affiliation(s) (note: participants could select all that apply)						
Lived Experience—Self	29	42.64%	23	19.5%	22	19.0%
Lived Experience—Other	53	77.94%	44	37.3%	43	37.2%
Service Delivery^a^	35	51.47%	26	22.0%	23	19.8%
Policy/Decision Maker	14	20.59%	10	8.5%	10	8.6%
Researcher	18	26.47%	15	12.7%	15	12.9%
Other	—	—	3	2.5%	3	2.6%

^a^ “Service Delivery” comprises clinical and non‐clinical professionals. 10/31 were clinical in round 1, 8/20 were clinical in rounds 2 and 3.

### 3.2 Delphi Process

**FIGURE 3:**
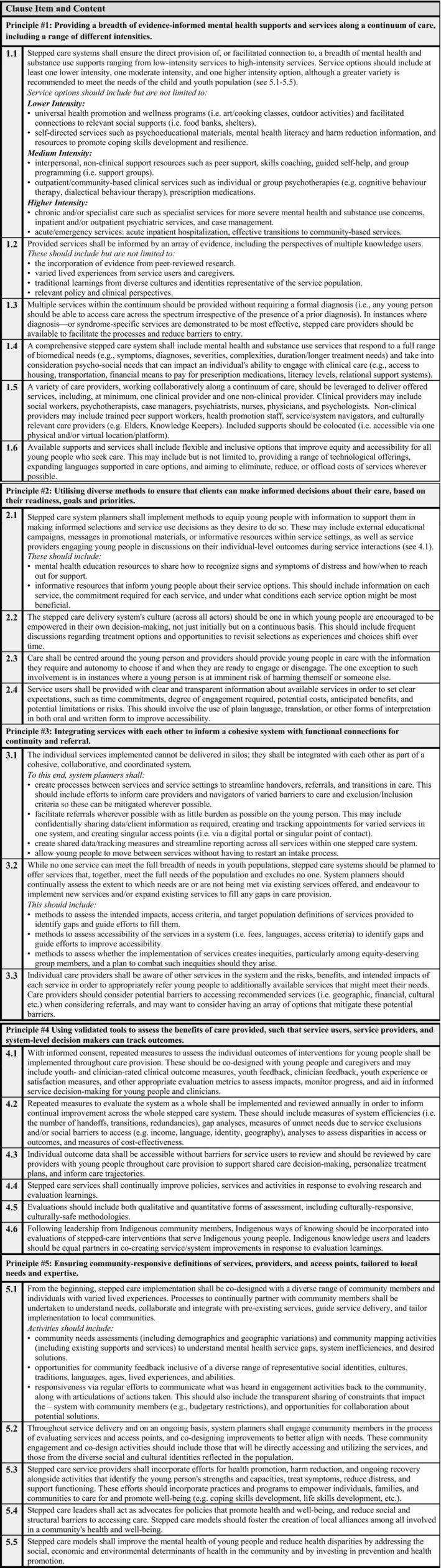
Full standard for implementing stepped care in child and youth mental health systems. *Note:* Final numbering of clauses does not align with Table 2; numbering of clauses was redone with the final list.

Over 3 Delphi rounds, 24 clause items were formulated across 5 guiding principles that comprised standards for implementing SC in CYMH settings (Figure 3). Of the original 29 clauses presented in Round 1 (Figure S2), 4 were included as initially written, 13 were included after Round 2, and 7 were included after Round 3. The remaining five were removed, and their content was incorporated into other clauses due to participant feedback (Table 2). For a detailed summary of consensus across stakeholder groups and Delphi rounds, see Tables S1–S3.

**TABLE 2:** Delphi results across rounds 1–3.

	Clause Item	Round 1				Round 2				Round 3			
		Step 1	Step 2	Step 3	Decision	Step 1	Step 2	Step 3	Decision	Step 1	Step 2	Step 3	Decision
1	1.1	Send to Revision		Clear Revisions	Send to Round 2	Tentative Pass	Sufficient Agreement	Pass		Passed in Round 2			
	1.2	Tentative Pass	Sufficient Agreement	Clear Revisions	Send to Round 2	Tentative Pass	Sufficient Agreement	Pass		Passed in Round 2			
	1.3	Send to Revision		Clear Revisions	Send to Round 2	Send to Revision		Clear Revisions	Send to Round 3	Tentative Pass	Sufficient Agreement	Pass	
	1.4	Send to Revision		Clear Revisions	Send to Round 2	Tentative Pass	Complete Agreement	Pass		Passed in Round 2			
	1.5	Send to Revision		Clear Revisions	Send to Round 2	Tentative Pass	Sufficient Agreement	Clear Revisions	Send to Round 3	Tentative Pass	Sufficient Agreement	Pass	
	1.6	Send to Revision		Clear Revisions	Send to Round 2	Tentative Pass	Sufficient Agreement	Pass		Passed in Round 2			
2	2.1	Send to Revision		Clear Revisions	Send to Round 2	Send to Revision		Clear Revisions	Send to Round 3	Tentative Pass	Sufficient Agreement	Pass	
	2.2	Tentative Pass	Complete Agreement	Pass		Passed in Round 1							
	2.3	Send to Revision		Clear Revisions	Send to Round 2	Tentative Pass	Sufficient Agreement	Pass		Passed in Round 2			
	2.4	Tentative Pass	Sufficient Agreement	Clear Revisions	Send to Round 2	Tentative Pass	Complete Agreement	Pass		Passed in Round 2			
	2.5	Send to Revision		Redundant	Remove	Removed in Round 1 (Redundant)				Removed in Round 2 (Redundant)			
3	3.1	Tentative Pass	Sufficient Agreement	Clear Revisions	Send to Round 2	Tentative Pass	Sufficient Agreement	Pass		Passed in Round 2			
	3.2	Send to Revision		Clear Revisions	Send to Round 2	Tentative Pass	Sufficient Agreement	Clear Revisions	Send to Round 3	Tentative Pass	Sufficient Agreement	Pass	
	3.3	Tentative Pass	Complete Agreement	Pass		Passed in Round 1				Passed in Round 1			
4	4.1	Tentative Pass	Sufficient Agreement	Clear Revisions	Send to Round 2	Tentative Pass	Sufficient Agreement	Clear Revisions	Send to Round 3	Tentative Pass	Sufficient Agreement	Pass	
	4.2	Tentative Pass	Insufficient Agreement	Clear Revisions	Send to Round 2	Tentative Pass	Insufficient Agreement	Clear Revisions	Send to Round 3	Tentative Pass	Insufficient Agreement	Pass^a^	
	4.3	Send to Revision		Clear Revisions	Send to Round 2	Tentative Pass	Sufficient Agreement	Clear Revisions	Send to Round 3	Tentative Pass	Complete Agreement	Pass	
	4.4	Send to Revision		Redundant	Remove	Removed in Round 1 (Redundant)							
	4.5	Send to Revision		Clear Revisions	Send to Round 2	Tentative Pass	Sufficient Agreement	Pass		Passed in Round 2			
	4.6	Tentative Pass	Insufficient Agreement	Redundant	Remove	Removed in Round 1 (Redundant)							
	4.7	Tentative Pass	Sufficient Agreement	Redundant	Remove	Removed in Round 1 (Redundant)							
	4.8	Tentative Pass	Complete Agreement	Pass		Passed in Round 1							
	4.9	Tentative Pass	Sufficient Agreement	Clear Revisions	Send to Round 2	Tentative Pass	Complete Agreement	Pass		Passed in Round 2			
	4.10	Send to Revision		Redundant	Remove	Removed in Round 1 (Redundant)							
5	5.1	Tentative Pass	Sufficient Agreement	Clear Revisions	Send to Round 2	Tentative Pass	Complete Agreement	Pass		Passed in Round 2			
	5.2	Tentative Pass	Sufficient Agreement	Clear Revisions	Send to Round 2	Tentative Pass	Complete Agreement	Pass		Passed in Round 2			
	5.3	Tentative Pass	Sufficient Agreement	Clear Revisions	Send to Round 2	Tentative Pass	Complete Agreement	Pass		Passed in Round 2			
	5.4	Tentative Pass	Sufficient Agreement	Clear Revisions	Send to Round 2	Tentative Pass	Complete Agreement	Pass		Passed in Round 2			
	5.5	Tentative Pass	Complete Agreement	Pass		Passed in Round 1							

^a^ While this item did not receive sufficient agreement, agreement was very high. Only one group voted < 70% agreement on 5 or 6, and this group was short of this threshold by one vote which was a 4; “Neutral, Leaning Agree”. Also, this respondent gave no comments to suggest revisions, and so this item was passed.

### 3.3 Themes Across Revisions

We received 701 comments across the 3 Delphi rounds. The most frequent category of comments were “Revision”, with 323 such comments received. Comments tagged as “Revision” were thematically coded and are presented in Table 3, including clarifying ambiguity (i.e., specify words like “multiple”), considering limitations of patient autonomy (e.g., when safety is at risk) and the need to clarify roles and responsibilities in system‐wide activities. These themes are expanded upon below.

**TABLE 3:** Comment codes by round.

Comment code	Round 1 comments		Round 2 comments		Round 3 comments	
	*N* = 468		*N* = 194		*N* = 39	
Revision	229	48.62%	84	42.42%	10	25.64%
No Guidance	88	18.68%	55	27.78%	9	23.08%
Approval	37	7.86%	17	8.59%	3	7.69%
Need for Glossary	27	5.73%	5	2.53%	2	5.13%
Implementation Idea	20	4.25%	1	0.51%	6	15.38%
Other	18	3.82%	2	1.01%	3	7.69%
Misunderstanding	13	2.76%	7	3.54%	2	5.13%
Question	12	2.55%	2	1.01%	2	5.13%
Translation Issue	9	1.91%	8	4.04%	2	5.13%
KT Question	8	1.70%	6	3.03%	0	0.00%
Process/Delphi Question	3	0.64%	0	0.00%	0	0.00%
Disagreement	3	0.64%	0	0.00%	0	0.00%
Content re: Other Clause	1	0.21%	7	3.54%	0	0.00%
Themes Across Revision Comment	Clarify ambiguity (i.e., multiple, various). Define accessibility/equity terms Improve language consistency (e.g., service user vs. client) Consistently incorporate substance use alongside mental health Consider the limitations of patient autonomy Need to clarify roles and responsibilities Enhance language of continual improvement		Greatly improved draft; revisions more minor Include more examples to demonstrate options Align language across principles and clauses Strengthen guidance around assessments and their role in guiding service delivery Single word choice recommendations		Define equity terms Single word choice recommendation	

#### 3.3.1 Clarifying Ambiguity

Respondents desired greater specificity in the guidance provided. For example, in Round 1, Clause 1.1 stated: “Stepped care systems shall ensure the direct provision of, or facilitated connection to, a breadth of mental health supports ranging from low‐intensity services to high‐intensity services.” This was followed by a bulleted list of service options to consider (Figure S2). Participants described this guidance as vague and lacking the necessary detail to inform implementation decisions. Comments included “It's unclear if this standard means that they have to include each of the things in the bulleted list,” and “Communities and their resources vary widely … perhaps this recommends 4 of 6 recommended services or something along those lines.”

This theme was also found in comments for Clause 1.5, which originally stated: “A variety of professionals should be leveraged to deliver included services. This may include social workers, trained peer support workers, psychotherapists, case managers, psychiatrists, nurses, physicians, and psychologists.” One respondent comment stated: “To me, this one is a bit vague. I agree with the sentiment but how much diversity do we mean (i.e., is 2 types of professionals enough? 3? 5?).”

Language across the standard was revised as a result of feedback to outline minimum requirements for a number of clauses (e.g., 1.1, 1.5) (Figure 3).

#### 3.3.2 Considering Limitations of Patient Autonomy

There was consensus around the need to implement methods that promote shared decision‐making between youth and service providers in SC systems, with clauses addressing this being among the first to achieve complete agreement (e.g., Clause 2.2). However, important questions were also raised about the limitations of client autonomy.

As an example, Clause 2.3 originally stated: “Care shall be centred around the service user and providers should provide service users with the autonomy to choose if and when they are ready to engage or disengage. Providers and family members may advocate for care options that they feel strongly will meet service user needs” (Figure S2). In response, one participant wrote: “As written it is unclear who is intended to have the final say. Do service users have full autonomy, or do providers and family have the final say in care options.” Others also pointed to exceptions to client autonomy, particularly where provider or family input might need to override youth preferences to protect safety. Based on these comments, Clause 2.3 was rewritten to clearly state exceptions to autonomy to protect safety (i.e., imminent suicidal ideation) (Figure 3).

In addition, questions around how much responsibility to place on young people were raised in general feedback provided. One respondent reflected, “[…] empowerment has become a way to place all the focus of care on the shoulders of people who are suffering while absolving the responsibility/role of supports.” In response, language to consistently promote shared decision‐making was strengthened throughout subsequent drafts to ensure that the responsibility of care is distributed across all those involved.

#### 3.3.3 Clarifying Roles and Responsibilities

Respondents queried which actors should hold the responsibility for ensuring service integration or system‐wide activities (e.g., evaluations). Specifically, in Round 1, Clause 3.1 stated: “The individual services implemented cannot be delivered in silos; they shall be integrated with each other as part of a cohesive and coordinated system.” This was complemented with a bulleted list of activities to consider for implementation (Figure S2). In response, one participant stated: “The system is designed to function in silos, so without a separate, dedicated provincial body to do the work of integration, I am not sure it will happen.”

In comments that were not specific to any individual clause, others wrote: “Who will ensure that all steps are being provided across agencies (assuming this is shared by agencies)? Who is accountable?”, and “I feel it is important to name what individuals should be in charge so that it is clear who has a role in what. If there are no clear guidelines on who does what, I fear things will never be followed.”

Efforts were made to clarify roles and responsibilities in revisions across the full standard between Delphi rounds. These included clarifications of roles, such as “Service providers should …” and “System planners should …” across multiple clause items where appropriate (Figure 3).

## 4 Discussion

The results of this study represent the first multi‐stakeholder, consensus‐driven set of standards for implementing SC in various CYMH settings across Canada. Given the significant variability in current SC models, these standards offer practical guidance for more consistent, evidence‐based implementation. Nevertheless, further efforts are necessary to facilitate their effective uptake. This may include the development of evaluation and fidelity scales, as well as the creation of additional resources to guide implementation in diverse contexts (i.e., case studies, plain‐language resources). In addition, this study also highlighted many areas that similarly require greater consensus, as many clauses outline practices that are, themselves, inconsistently defined in research and practice. Additional research is needed to strengthen alignment in these specific areas, including methods for effective community co‐design, shared decision‐making, measurement‐based care, and incorporating Indigenous ways of knowing into evaluations.

Moreover, a critical tension that surfaced during this study was balancing aspirational ideals with practical realities. Like most standards, these standards are inherently aspirational as they outline a transformative new model for delivering mental health care. To guide their creation, we asked participants to reflect on what care *should* look like in an ideal world. However, many participants face resource limitations and entrenched service silos that are difficult to overcome. In this context, it became essential to remind participants that perceived challenges pertaining to implementing these standards should not compromise the creation of an “ideal” standard. It will now be critical for future research to further explore how aspirational standards can be effectively operationalised in constrained environments. This should include examinations of the barriers and facilitators to their implementation and work to adapt these standards to different contexts (e.g., rural vs. urban settings, culturally diverse adaptations).

This study also uncovered unresolved, system‐wide questions. Where child‐ and youth‐specific mental health systems exist, they are often disjointed and complex, frequently lacking a single governing authority accountable for their structure, implementation, and coordination. Much of the guidance outlined in this standard requires substantial, cross‐system leadership to operationalise (e.g., evaluating system‐level outcomes, ensuring smooth transitions between services, and fostering ongoing community engagement). As a result, many respondents raised the question of who would, or could, be responsible for this work.

It is our hope that this standard will not only guide the development of stronger systems, but will also serve as a catalyst for significant reforms and emerging systems‐focused leadership. Examples of this have already been demonstrated by the uptake of SC across systems of care. Integrated youth services have created a novel integrated SC model to better coordinate youth mental health services across the province of British Columbia (Foundry 2023). Similarly, several post‐secondary institutions have leveraged SC to build more cohesive mental health systems for their students (Cornish et al. 2017). In addition, individual services can leverage this standard to spark micro‐level system improvements; improving coordination both within their own services and across other locally available resources (e.g., assessing how they complement or overlap with other locally available services, considering partnership opportunities for integration and referral).

In summary, while SC models offer a framework to create cohesive mental health systems that fundamentally differ from those typically present in Canada, they also offer an opportunity to innovate and commit to the creation of service enhancements that connect—rather than fragment—our systems of care.

### 4.1 Limitations

First, this study was based in Canada with experts representing Canadian mental health care delivery and lived experience; as a result, guidance outlined in this standard may not be relevant in international settings. Second, this standard was not designed with any individual community in mind. As such, future efforts to apply and adapt this standard to specific contexts and demographic groups, including but not limited to Indigenous, rural, and Northern settings, may be needed to create context‐dependent and culturally responsive SC systems. Third, this standard does not outline methods for its evaluation or quality improvement. Future work will include efforts to operationalise and consider applying these standards to specific settings (Objective 3) and to measure individual and systemic outcomes.

## 5 Conclusion

Standards, by definition, are aspirational; they set a benchmark for us to aim to achieve, or even surpass, when designing care provided (in this case) to children and youth. Standards are nonetheless critically important. Despite resourcing and other feasibility‐related challenges, they are needed in order to align care around best practices and outline areas where flexibility is appropriate (e.g., business hours, selected clinical impact measures) as well as areas of inflexibility (i.e., the availability of acute mental health services and specialist care providers). This standard represents a consensus‐built list of elements that ought to be in place to build cohesive mental health systems that meet the needs of all of Canada's children and youth.

## Conflicts of Interest

Co‐author Dr. AnnMarie Churchill is employed by Stepped Care Solutions, the Stepped Care 2.0 model developer. The other authors declare no conflicts of interest.

## Supporting information


Data S1.

## Data Availability

Data available on request from the authors.
