# Supplementary material for: Proposed Standards for Implementing Stepped Care Models in Child and Youth Mental Health Service Systems: Results of a Pan‐Canadian Delphi Study
Source: Early Interv Psychiatry. 2025 May 30;19(6):e70057. doi: 10.1111/eip.70057 (PMC12124162; doi:10.1111/eip.70057)
Supplement: Supplementary file 1 — Data S1. [file EIP-19-0-s001.docx]

**SUPPLEMENTAL TABLES & FIGURES**

**Supplemental Figure 1: Example of Clause Revision from Round 1-3**

| **Principle 1:**  Provide a breadth of evidence-informed mental health supports and services along a continuum of care, including a range of different intensities. | | |
| --- | --- | --- |
| **Round 1** | **Clause Item 1.5, Original Draft:**  A variety of professionals should be leveraged to deliver included services. This may include social workers, trained peer support workers, psychotherapists, case managers, psychiatrists, nurses, physicians, and psychologists. Included supports may be colocated or provided by a variety of different professionals in different settings as required. | |
|  | **Round Result:** | **Step 1 of the Decision Support Tool (Figure 2)**  Item did not reach >70% group consensus. Send to Revision Pool. |
|  | **Revisions:** | **Step 3 of the Decision Support Tool (Figure 2)**  Comments received during Delphi Round #1 specific to 1.5 were synthesised and discussed by the Research Team (RT):   - *revise "professionals";* - *include low-intensity or informal supports;* - *include cultural service providers/supports;* - *clarify number required per tier;* - *consider revising co-location* |
| **Round 2** | **Clause Item 1.5, Revised for Delphi Round 2:**  A variety of service providers should be leveraged to deliver included services including, at minimum, one clinical provider and one non-clinical provider. Clinical providers may include social workers, psychotherapists, case managers, psychiatrists, nurses, physicians, and psychologists. Non-clinical providers may include trained peer support workers, health promotion staff, and culturally-relevant care providers. Included supports may be colocated (i.e. accessible via one location) or can be provided by a variety of integrated professionals in different settings as required. | |
|  | **Round Result:** | **Step 1 of the Decision Support Tool (Figure 2)** Tentative Pass. Item received >70% group consensus. |
|  |  | **Step 2 of the Decision Support Tool (Figure 2)** Sufficient Agreement. Item received >70% consensus across all stakeholder groups. |
|  | **Revisions:** | **Step 3 of the Decision Support Tool (Figure 2)**  Comments received during Delphi Round #2 were synthesised and discussed by the RT. Although this item received sufficient agreement across all groups, the RT felt that the comments received helped to further clarify the clause and opted to include it in Round #3.   - *note that colocation can be in-person/online;* - *incorporate more prevention service examples;* - *introduce concept of collaborative care* |
| **Round 3** | **Clause Item 1.5, Revised for Delphi Round 3:**  A variety of care providers, working collaboratively along a continuum of care, should be leveraged to deliver offered services, including, at minimum, one clinical provider and one non-clinical provider. Clinical providers may include social workers, psychotherapists, case managers, psychiatrists, nurses, physicians, and psychologists. Non-clinical providers may include trained peer support workers, health promotion staff, service/system navigators, and culturally relevant care providers (e.g. Elders, Knowledge Keepers). Included supports should be colocated (i.e. accessible via one physical and/or virtual location/platform) | |
|  | **Round Result:** | **Step 1 of the Decision Support Tool (Figure 2)** Tentative Pass. Item received >70% group consensus. |
|  |  | **Step 2 of the Decision Support Tool (Figure 2)** Sufficient Agreement. Item received >70% consensus across all stakeholder groups. |
|  | **Revisions:** | **Step 3 of the Decision Support Tool (Figure 2)**  No further comments received. Item was passed to the final standard list. |

**Supplemental Figure 2:**

**Initial Draft Standard for Implementing Stepped Care in Child and Youth Mental Health Systems**

(Used in Round 1 of Delphi)

| **Clause Item and Content** | |
| --- | --- |
| **Principle #1: Providing a breadth of evidence-informed mental health supports and services along a continuum of care, including a range of different intensities.** | |
| **1.1** | Stepped care systems shall ensure the direct provision of, or facilitated connection to, a breadth of mental health supports ranging from low-intensity services to high-intensity services.  *Provided services should include:*   - informal services such as health promotion and wellness programs (i.e. art/cooking classes, outdoor activities). - self-directed services such as psychoeducational materials, mental health literacy information, and resources to promote coping skills development and resilience. - interpersonal, non-clinical support resources such as peer support, skills coaching, guided self-help, and group programming (i.e. support groups). - outpatient/community-based clinical services such as individual or group psychotherapies (e.g. cognitive behaviour therapy, dialectical behaviour therapy), prescription medications. - chronic and/or specialist care such as specialist services for more severe mental health and substance use concerns, inpatient and/or outpatient psychiatric services, case management. - acute/emergency services: acute inpatient hospitalization, effective transitions to community-based services. |
| **1.2** | Provided services shall be informed by an array of evidence including the perspectives of multiple stakeholders  *This should include:*   - the incorporation of evidence from peer-reviewed research. - traditional learnings from diverse cultures and identities representative of the service population. - varied lived experiences from service users and caregivers. |
| **1.3** | Multiple provided services within the continuum should be transdiagnostic (i.e. provided without exclusionary diagnostic criteria or categorizations), allowing service users to access the full range of supports across the continuum. |
| **1.4** | Sufficient breadth of services shall be provided such that no one is turned away. A comprehensive stepped care system shall include services to respond to a full range of diagnoses, severity, complexity, and acuity. |
| **1.5** | A variety of professionals should be leveraged to deliver included services. This may include social workers, trained peer support workers, psychotherapists, case managers, psychiatrists, nurses, physicians, and psychologists. Included supports may be colocated or provided by a variety of different professionals in different settings as required. |
| **1.6** | Available supports and services shall include options that are equitable, diverse, inclusive and accessible to all service users. To do so, this may include offerings delivered in a range of modalities such as in-person or virtual settings, and/or within health care or via community partnerships. |
| **Principle #2: Utilising diverse methods to ensure that clients can make informed decisions about their care, based on their readiness, goals and priorities.** | |
| **2.1** | Stepped care system planners shall implement methods to equip the service's target population with information to make informed selections and service use decisions.  *This should include:*   - mental health literacy resources to teach the target population to recognize signs and symptoms of distress and how/when to reach out for support. These may include external campaigns or simply resources within service settings. - informative resources that teach the target population about their service options. This should include information on each service, the commitment required for each service, and under what conditions each service option might be most beneficial. These may include external campaigns or simply resources within service settings. - service providers across the full system are trained to inform clients of their service options, to engage clients in conversations to support client decision making and to enable clients to consider their readiness, preferences, and ability to invest time and resources. |
| **2.2** | The stepped care delivery system's culture (across all actors) should be one in which clients are encouraged to be empowered in their own decision-making, not just initially but on a continuous basis. This should include frequent discussions regarding treatment options and opportunities to revisit selections as experiences and choices shift over time. |
| **2.3** | Care shall be centred around the service user and providers should provide service users with the autonomy to choose if and when they are ready to engage or disengage. Providers and family members may advocate for care options that they feel strongly will meet service user needs. |
| **2.4** | Service users shall be provided with clear and transparent information about available services in order to set clear expectations, such as time commitments, degree of engagement required, anticipated benefits, and potential limitations or risks. This may involve the use of plain language, translation, or other forms of interpretation in both oral and written form. |
| **2.5** | Individuals who engage service users along their care trajectory shall make their assumptions explicit in order to ensure that service users have sufficient information to make truly informed decisions. |
| **Principle #3: Integrating services with each other to inform a cohesive system with functional connections for continuity and referral.** | |
| **3.1** | The individual services implemented cannot be delivered in silos; they shall be integrated with each other as part of a cohesive and coordinated system.  *To this end, system planners shall:*   - create processes between services and service settings to streamline handovers, referrals, and transitions in care. - facilitate referrals wherever possible with as little burden as possible on the client. This may include confidentially sharing data/client information as required, creating and tracking appointments for varied services in one system, and creating singular access points (i.e. via a digital portal or singular point of contact). - create shared data/tracking measures and streamline reporting across all services within one stepped care system. - allow clients to move between services without having to restart an intake process. |
| **3.2** | Services implemented shall be planned with consideration to their broader role within the larger system.  *This shall include:*   - methods to assess the intended impacts and target populations of services provided within a stepped care system to identify gaps and guide the design of new services. - methods to assess accessibility of the services in a system (i.e. fees, languages, criteria) and complement less accessible services with accessible options - methods to assess whether the implementation of services creates inequities, particularly among equity-seeking group members, and a plan to combat such inequalities should they arise. |
| **3.3** | Individual service providers shall be aware of other services in the system and the risks, benefits, and intended impacts of each service in order to appropriately refer service users to additionally available services that might meet their needs. Service providers should consider potential barriers to accessing recommended services (i.e. geographic, financial, cultural etc.) when considering referrals, and may want to consider having an array of options that mitigate these potential barriers. |
| **Principle #4 Using validated tools to assess the benefits of care provided, such that service users, service providers, and system-level decision makers can track outcomes.** | |
| **4.1** | Measures to assess the individual benefits of interventions for clients shall be regularly implemented. These should include clinical outcome measures, client feedback, clinician feedback, client experience or satisfaction measures, and other appropriate evaluation metrics to assess impacts, guide service decisions, monitor progress, and aid in informed decision-making for clients and clinicians. |
| **4.2** | System measures to assess the system as a whole shall be regularly implemented in order to inform continual improvement across the whole stepped care system. These should include measures of cost-effectiveness, system efficiencies (i.e. number of handoffs, transitions, redundancies), gap analyses, and measures of unmet needs. |
| **4.3** | Service data shall be regularly reviewed by service providers and service users in the spirit of shared care decision making to personalize treatment and inform care trajectories. |
| **4.4** | Data from routinely collected outcomes shall be owned by the service user. |
| **4.5** | Stepped care services shall adapt policies, services and activities in accordance with evaluation outcomes. All delivered services should have recursive mechanisms for assessment and clearly articulated plans for ongoing evaluation and continual improvement. |
| **4.6** | Evaluation of stepped care interventions should use participatory approaches that meaningfully involve all care partners, including patients/clients and community representatives. |
| **4.7** | Evaluations of stepped-care interventions should include a variety of clinical and non-clinical measures focussed on outcomes for individual patients/clients, families, communities, and participating service systems |
| **4.8** | Evaluations should include both qualitative and quantitative forms of assessment, including culturally-responsive, culturally-safe methodologies. |
| **4.9** | Indigenous ways of knowing should be incorporated into evaluations of stepped-care interventions that serve Indigenous patients/clients. |
| **4.10** | Data pertaining to stepped-care services shall have a mechanism that provides routine evaluation with a lens for social accountability. |
| **Principle #5: Ensuring community-responsive definitions of services, providers, and access points, tailored to local needs and expertise.** | |
| **5.1** | Processes to continually engage with diverse community members shall be undertaken to understand needs, adapt service offerings, and tailor stepped care systems to local communities.  These should include:   - community needs assessments (including demographics and geographic variations) and community mapping activities (including existing supports and services) to understand mental health service gaps, system inefficiencies, and desired solutions. - opportunities for diverse community feedback inclusive of representative identities, cultures, traditions, languages, ages, lived experiences, and abilities. - responsiveness via regular efforts to communicate what was heard in engagement activities back to the community, along with articulations of actions taken. This should also include the transparent sharing of constraints that impact the – system with community members (e.g., budgetary restrictions), and opportunities for collaboration about potential solutions. |
| **5.2** | System planners shall engage communities in the process of co-designing services and access points, with a process for updating regularly. These co-design activities should include those that will be directly accessing and utilizing the services. |
| **5.3** | Stepped care services shall be built on practices that empower individuals, families, and communities to optimize their health: as advocates for policies that promote and protect health and well-being, as co-developers of health and social services, and as self-carers and caregivers.^a^ |
| **5.4** | Stepped care models should foster the creation of local alliances among all involved in a community's health and well-being. Stepped care models should articulate a shared vision and common goals, and partnerships should actively strive for balanced power relations and mutual trust.^a^ |
| **5.5** | Stepped care models shall improve the mental health of young people and reduce health disparities by addressing the social, economic and environmental determinants of health in the community and by investing in prevention and health promotion.^a^ |

^a^ adapted from Transform Integrated Community Care.Integrated Community Care 4All Seven Principles for Care. 2020. https://transform-integratedcommunitycare.com/wp-content/uploads/2021/03/ICC4All-Strategy_EN.pdf

Note: Initial numbering of clauses does not align with final numbering in Figure 3; numbering of clauses was redone with the final list.

**Supplemental Table 1:**

**Round 1 Likert Responses by Clause Item and by Stakeholder Group**

| **Clause**  **Item** | **Stakeholder**  **Group** | **Responses** | | | | | | **Score**  **Decision** | **Review**  **Decision** |
| --- | --- | --- | --- | --- | --- | --- | --- | --- | --- |
|  |  | **1** | **2** | **3** | **4** | **5** | **6** |  |  |
| **1.1** | Lived Experience, Self | 0.00% | 0.00% | 0.00% | 0.00% | 25.00% | 75.00% | Sufficient Agreement - May Pass; Review Comments | Revise |
|  | Lived Experience, Other | 0.00% | 0.00% | 0.00% | 0.00% | 16.67% | 83.33% |  |  |
|  | Service Delivery | 0.00% | 0.00% | 0.00% | 0.00% | 50.00% | 50.00% |  |  |
|  | Policy/Decision Maker | 0.00% | 0.00% | 0.00% | 0.00% | 50.00% | 50.00% |  |  |
|  | Researcher | 0.00% | 0.00% | 0.00% | 0.00% | 41.94% | 58.06% |  |  |
| **1.2** | Lived Experience, Self | 0.00% | 0.00% | 8.33% | 0.00% | 8.33% | 83.33% | Sufficient Agreement - May Pass; Review Comments | Revise |
|  | Lived Experience, Other | 0.00% | 0.00% | 0.00% | 0.00% | 8.33% | 91.67% |  |  |
|  | Service Delivery | 0.00% | 0.00% | 0.00% | 0.00% | 50.00% | 50.00% |  |  |
|  | Policy/Decision Maker | 0.00% | 0.00% | 0.00% | 0.00% | 41.67% | 58.33% |  |  |
|  | Researcher | 0.00% | 0.00% | 0.00% | 6.25% | 28.12% | 65.62% |  |  |
| **1.3** | Lived Experience, Self | 0.00% | 0.00% | 0.00% | 9.09% | 36.36% | 54.55% | Sufficient Agreement - May Pass; Review Comments | Revise |
|  | Lived Experience, Other | 0.00% | 0.00% | 0.00% | 0.00% | 8.33% | 91.67% |  |  |
|  | Service Delivery | 0.00% | 0.00% | 0.00% | 0.00% | 50.00% | 50.00% |  |  |
|  | Policy/Decision Maker | 0.00% | 8.33% | 8.33% | 8.33% | 58.33% | 16.67% |  |  |
|  | Researcher | 3.12% | 3.12% | 6.25% | 6.25% | 28.12% | 53.12% |  |  |
| **1.4** | Lived Experience, Self | 0.00% | 0.00% | 0.00% | 0.00% | 9.09% | 90.91% | Sufficient Agreement - May Pass; Review Comments | Revise |
|  | Lived Experience, Other | 0.00% | 0.00% | 0.00% | 0.00% | 16.67% | 83.33% |  |  |
|  | Service Delivery | 0.00% | 0.00% | 0.00% | 0.00% | 50.00% | 50.00% |  |  |
|  | Policy/Decision Maker | 0.00% | 0.00% | 8.33% | 8.33% | 16.67% | 66.67% |  |  |
|  | Researcher | 0.00% | 3.23% | 3.23% | 3.23% | 32.26% | 58.06% |  |  |
| **1.5** | Lived Experience, Self | 0.00% | 0.00% | 0.00% | 0.00% | 9.09% | 90.91% | Sufficient Agreement - May Pass; Review Comments | Revise |
|  | Lived Experience, Other | 0.00% | 0.00% | 0.00% | 0.00% | 33.33% | 66.67% |  |  |
|  | Service Delivery | 0.00% | 0.00% | 0.00% | 0.00% | 50.00% | 50.00% |  |  |
|  | Policy/Decision Maker | 0.00% | 8.33% | 8.33% | 8.33% | 41.67% | 33.33% |  |  |
|  | Researcher | 0.00% | 0.00% | 3.23% | 9.68% | 22.58% | 64.52% |  |  |
| **1.6** | Lived Experience, Self | 0.00% | 0.00% | 0.00% | 0.00% | 36.36% | 63.64% | Sufficient Agreement - May Pass; Review Comments | Revise |
|  | Lived Experience, Other | 0.00% | 0.00% | 0.00% | 0.00% | 8.33% | 91.67% |  |  |
|  | Service Delivery | 0.00% | 0.00% | 0.00% | 0.00% | 0.00% | 100.00% |  |  |
|  | Policy/Decision Maker | 0.00% | 0.00% | 16.67% | 8.33% | 16.67% | 58.33% |  |  |
|  | Researcher | 0.00% | 0.00% | 0.00% | 9.68% | 29.03% | 61.29% |  |  |
| **2.1** | Lived Experience, Self | 0.00% | 0.00% | 0.00% | 18.18% | 36.36% | 45.45% | Sufficient Agreement - May Pass; Review Comments | Revise |
|  | Lived Experience, Other | 0.00% | 0.00% | 0.00% | 0.00% | 8.33% | 91.67% |  |  |
|  | Service Delivery | 0.00% | 0.00% | 0.00% | 0.00% | 0.00% | 100.00% |  |  |
|  | Policy/Decision Maker | 0.00% | 0.00% | 0.00% | 9.09% | 63.64% | 27.27% |  |  |
|  | Researcher | 0.00% | 0.00% | 0.00% | 3.23% | 35.48% | 61.29% |  |  |
| **2.2** | Lived Experience, Self | 0.00% | 9.09% | 0.00% | 0.00% | 18.18% | 72.73% | Complete Agreement - Pass | Pass |
|  | Lived Experience, Other | 0.00% | 0.00% | 0.00% | 0.00% | 16.67% | 83.33% |  |  |
|  | Service Delivery | 0.00% | 0.00% | 0.00% | 0.00% | 0.00% | 100.00% |  |  |
|  | Policy/Decision Maker | 0.00% | 0.00% | 0.00% | 0.00% | 27.27% | 72.73% |  |  |
|  | Researcher | 0.00% | 0.00% | 0.00% | 3.23% | 22.58% | 74.19% |  |  |
| **2.3** | Lived Experience, Self | 0.00% | 0.00% | 9.09% | 0.00% | 18.18% | 72.73% | Sufficient Agreement - May Pass; Review Comments | Revise |
|  | Lived Experience, Other | 0.00% | 8.33% | 0.00% | 0.00% | 8.33% | 83.33% |  |  |
|  | Service Delivery | 0.00% | 0.00% | 0.00% | 0.00% | 50.00% | 50.00% |  |  |
|  | Policy/Decision Maker | 0.00% | 0.00% | 0.00% | 0.00% | 63.64% | 36.36% |  |  |
|  | Researcher | 0.00% | 0.00% | 3.23% | 3.23% | 38.71% | 54.84% |  |  |
| **2.4** | Lived Experience, Self | 0.00% | 0.00% | 0.00% | 0.00% | 27.27% | 72.73% | Sufficient Agreement - May Pass; Review Comments | Revise |
|  | Lived Experience, Other | 0.00% | 0.00% | 0.00% | 0.00% | 16.67% | 83.33% |  |  |
|  | Service Delivery | 0.00% | 0.00% | 0.00% | 0.00% | 0.00% | 100.00% |  |  |
|  | Policy/Decision Maker | 0.00% | 0.00% | 18.18% | 9.09% | 18.18% | 54.55% |  |  |
|  | Researcher | 0.00% | 0.00% | 0.00% | 0.00% | 29.03% | 70.97% |  |  |
| **2.5** | Lived Experience, Self | 0.00% | 18.18% | 0.00% | 9.09% | 27.27% | 45.45% | Insufficient Agreement - 1+ Group Disagrees - Revise | Remove; incorporated into another clause |
|  | Lived Experience, Other | 0.00% | 8.33% | 8.33% | 16.67% | 41.67% | 25.00% |  |  |
|  | Service Delivery | 0.00% | 0.00% | 0.00% | 50.00% | 0.00% | 50.00% |  |  |
|  | Policy/Decision Maker | 0.00% | 0.00% | 27.27% | 36.36% | 9.09% | 27.27% |  |  |
|  | Researcher | 6.45% | 12.90% | 6.45% | 9.68% | 25.81% | 38.71% |  |  |
| **3.1** | Lived Experience, Self | 0.00% | 0.00% | 0.00% | 0.00% | 9.09% | 90.91% | Sufficient Agreement - May Pass; Review Comments | Revise |
|  | Lived Experience, Other | 0.00% | 0.00% | 0.00% | 0.00% | 8.33% | 91.67% |  |  |
|  | Service Delivery | 0.00% | 0.00% | 0.00% | 0.00% | 50.00% | 50.00% |  |  |
|  | Policy/Decision Maker | 0.00% | 0.00% | 0.00% | 0.00% | 27.27% | 72.73% |  |  |
|  | Researcher | 0.00% | 3.23% | 0.00% | 3.23% | 25.81% | 67.74% |  |  |
| **3.2** | Lived Experience, Self | 0.00% | 0.00% | 0.00% | 0.00% | 45.45% | 54.55% | Sufficient Agreement - May Pass; Review Comments | Revise |
|  | Lived Experience, Other | 0.00% | 0.00% | 0.00% | 0.00% | 8.33% | 91.67% |  |  |
|  | Service Delivery | 0.00% | 0.00% | 0.00% | 0.00% | 0.00% | 100.00% |  |  |
|  | Policy/Decision Maker | 0.00% | 0.00% | 0.00% | 27.27% | 27.27% | 45.45% |  |  |
|  | Researcher | 0.00% | 0.00% | 0.00% | 0.00% | 35.48% | 64.52% |  |  |
| **3.3** | Lived Experience, Self | 0.00% | 0.00% | 0.00% | 0.00% | 18.18% | 81.82% | Sufficient Agreement - May Pass; Review Comments | Pass |
|  | Lived Experience, Other | 0.00% | 0.00% | 0.00% | 0.00% | 25.00% | 75.00% |  |  |
|  | Service Delivery | 0.00% | 0.00% | 0.00% | 0.00% | 0.00% | 100.00% |  |  |
|  | Policy/Decision Maker | 0.00% | 0.00% | 9.09% | 9.09% | 9.09% | 72.73% |  |  |
|  | Researcher | 0.00% | 0.00% | 0.00% | 12.90% | 16.13% | 70.97% |  |  |
| **4.1** | Lived Experience, Self | 0.00% | 0.00% | 0.00% | 9.09% | 9.09% | 81.82% | Sufficient Agreement - May Pass; Review Comments | Revise |
|  | Lived Experience, Other | 0.00% | 0.00% | 0.00% | 8.33% | 8.33% | 83.33% |  |  |
|  | Service Delivery | 0.00% | 0.00% | 0.00% | 0.00% | 0.00% | 100.00% |  |  |
|  | Policy/Decision Maker | 0.00% | 0.00% | 9.09% | 9.09% | 18.18% | 63.64% |  |  |
|  | Researcher | 0.00% | 0.00% | 0.00% | 9.68% | 22.58% | 67.74% |  |  |
| **4.2** | Lived Experience, Self | 0.00% | 0.00% | 0.00% | 0.00% | 18.18% | 81.82% | Insufficient Agreement - 1+ Group Disagrees - Revise | Revise |
|  | Lived Experience, Other | 0.00% | 0.00% | 0.00% | 0.00% | 8.33% | 91.67% |  |  |
|  | Service Delivery | 0.00% | 0.00% | 0.00% | 50.00% | 0.00% | 50.00% |  |  |
|  | Policy/Decision Maker | 0.00% | 0.00% | 9.09% | 18.18% | 27.27% | 45.45% |  |  |
|  | Researcher | 0.00% | 0.00% | 0.00% | 3.23% | 16.13% | 80.65% |  |  |
| **4.3** | Lived Experience, Self | 0.00% | 0.00% | 0.00% | 0.00% | 27.27% | 72.73% | Insufficient Agreement - 1+ Group Disagrees - Revise | Revise |
|  | Lived Experience, Other | 0.00% | 8.33% | 8.33% | 0.00% | 16.67% | 66.67% |  |  |
|  | Service Delivery | 0.00% | 0.00% | 0.00% | 0.00% | 0.00% | 100.00% |  |  |
|  | Policy/Decision Maker | 0.00% | 0.00% | 0.00% | 36.36% | 18.18% | 45.45% |  |  |
|  | Researcher | 0.00% | 0.00% | 3.23% | 6.45% | 19.35% | 70.97% |  |  |
| **4.4** | Lived Experience, Self | 0.00% | 0.00% | 0.00% | 18.18% | 27.27% | 54.55% | Insufficient Agreement -  1+ Group Disagrees - Revise | Remove; incorporated into another clause |
|  | Lived Experience, Other | 0.00% | 0.00% | 16.67% | 25.00% | 8.33% | 50.00% |  |  |
|  | Service Delivery | 0.00% | 0.00% | 0.00% | 0.00% | 50.00% | 50.00% |  |  |
|  | Policy/Decision Maker | 0.00% | 9.09% | 18.18% | 18.18% | 36.36% | 18.18% |  |  |
|  | Researcher | 3.23% | 6.45% | 6.45% | 16.13% | 9.68% | 58.06% |  |  |
| **4.5** | Lived Experience, Self | 0.00% | 0.00% | 0.00% | 0.00% | 18.18% | 81.82% | Insufficient Agreement - 1+ Group Disagrees - Revise | Revise |
|  | Lived Experience, Other | 0.00% | 0.00% | 0.00% | 0.00% | 8.33% | 91.67% |  |  |
|  | Service Delivery | 0.00% | 0.00% | 0.00% | 50.00% | 0.00% | 50.00% |  |  |
|  | Policy/Decision Maker | 0.00% | 0.00% | 0.00% | 18.18% | 54.55% | 27.27% |  |  |
|  | Researcher | 0.00% | 0.00% | 3.23% | 3.23% | 29.03% | 64.52% |  |  |
| **4.6** | Lived Experience, Self | 0.00% | 0.00% | 0.00% | 0.00% | 18.18% | 81.82% | Insufficient Agreement - 1+ Group Disagrees - Revise | Remove; incorporated into another clause |
|  | Lived Experience, Other | 0.00% | 8.33% | 0.00% | 0.00% | 8.33% | 83.33% |  |  |
|  | Service Delivery | 0.00% | 0.00% | 0.00% | 50.00% | 0.00% | 50.00% |  |  |
|  | Policy/Decision Maker | 0.00% | 0.00% | 0.00% | 0.00% | 36.36% | 63.64% |  |  |
|  | Researcher | 0.00% | 0.00% | 3.23% | 3.23% | 12.90% | 80.65% |  |  |
| **4.7** | Lived Experience, Self | 0.00% | 0.00% | 0.00% | 0.00% | 18.18% | 81.82% | Sufficient Agreement - May Pass; Review Comments | Remove; incorporated into another clause |
|  | Lived Experience, Other | 0.00% | 0.00% | 0.00% | 0.00% | 8.33% | 91.67% |  |  |
|  | Service Delivery | 0.00% | 0.00% | 0.00% | 0.00% | 0.00% | 100.00% |  |  |
|  | Policy/Decision Maker | 0.00% | 0.00% | 0.00% | 18.18% | 36.36% | 45.45% |  |  |
|  | Researcher | 0.00% | 3.23% | 3.23% | 0.00% | 9.68% | 83.87% |  |  |
| **4.8** | Lived Experience, Self | 0.00% | 0.00% | 0.00% | 0.00% | 18.18% | 81.82% | Complete Agreement - Pass | Pass |
|  | Lived Experience, Other | 0.00% | 0.00% | 0.00% | 8.33% | 8.33% | 83.33% |  |  |
|  | Service Delivery | 0.00% | 0.00% | 0.00% | 0.00% | 0.00% | 100.00% |  |  |
|  | Policy/Decision Maker | 0.00% | 0.00% | 0.00% | 0.00% | 27.27% | 72.73% |  |  |
|  | Researcher | 0.00% | 3.23% | 3.23% | 3.23% | 12.90% | 77.42% |  |  |
| **4.9** | Lived Experience, Self | 9.09% | 0.00% | 0.00% | 0.00% | 27.27% | 63.64% | Sufficient Agreement - May Pass; Review Comments | Revise |
|  | Lived Experience, Other | 0.00% | 0.00% | 8.33% | 0.00% | 16.67% | 75.00% |  |  |
|  | Service Delivery | 0.00% | 0.00% | 0.00% | 0.00% | 0.00% | 100.00% |  |  |
|  | Policy/Decision Maker | 0.00% | 0.00% | 0.00% | 0.00% | 27.27% | 72.73% |  |  |
|  | Researcher | 3.23% | 0.00% | 0.00% | 0.00% | 12.90% | 83.87% |  |  |
| **4.10** | Lived Experience, Self | 9.09% | 0.00% | 0.00% | 0.00% | 27.27% | 63.64% | Insufficient Agreement - 1+ Group Disagrees - Revise | Remove; incorporated into another clause |
|  | Lived Experience, Other | 0.00% | 0.00% | 8.33% | 0.00% | 16.67% | 75.00% |  |  |
|  | Service Delivery | 0.00% | 0.00% | 0.00% | 0.00% | 0.00% | 100.00% |  |  |
|  | Policy/Decision Maker | 0.00% | 0.00% | 0.00% | 0.00% | 27.27% | 72.73% |  |  |
|  | Researcher | 3.23% | 0.00% | 0.00% | 0.00% | 12.90% | 83.87% |  |  |
| **5.1** | Lived Experience, Self | 0.00% | 0.00% | 0.00% | 0.00% | 18.18% | 81.82% | Sufficient Agreement - May Pass; Review Comments | Revise |
|  | Lived Experience, Other | 0.00% | 0.00% | 0.00% | 0.00% | 0.00% | 100.00% |  |  |
|  | Service Delivery | 0.00% | 0.00% | 0.00% | 0.00% | 0.00% | 100.00% |  |  |
|  | Policy/Decision Maker | 0.00% | 0.00% | 0.00% | 0.00% | 45.45% | 54.55% |  |  |
|  | Researcher | 0.00% | 0.00% | 0.00% | 3.23% | 12.90% | 83.87% |  |  |
| **5.2** | Lived Experience, Self | 0.00% | 9.09% | 0.00% | 0.00% | 0.00% | 90.91% | Sufficient Agreement - May Pass; Review Comments | Revise |
|  | Lived Experience, Other | 0.00% | 0.00% | 0.00% | 8.33% | 33.33% | 58.33% |  |  |
|  | Service Delivery | 0.00% | 0.00% | 0.00% | 0.00% | 0.00% | 100.00% |  |  |
|  | Policy/Decision Maker | 0.00% | 0.00% | 0.00% | 10.00% | 40.00% | 50.00% |  |  |
|  | Researcher | 0.00% | 3.23% | 0.00% | 3.23% | 12.90% | 80.65% |  |  |
| **5.3** | Lived Experience, Self | 0.00% | 0.00% | 0.00% | 0.00% | 9.09% | 90.91% | Sufficient Agreement - May Pass; Review Comments | Revise |
|  | Lived Experience, Other | 0.00% | 0.00% | 0.00% | 0.00% | 0.00% | 100.00% |  |  |
|  | Service Delivery | 0.00% | 0.00% | 0.00% | 0.00% | 50.00% | 50.00% |  |  |
|  | Policy/Decision Maker | 0.00% | 0.00% | 0.00% | 0.00% | 45.45% | 54.55% |  |  |
|  | Researcher | 0.00% | 3.23% | 0.00% | 12.90% | 16.13% | 67.74% |  |  |
| **5.4** | Lived Experience, Self | 0.00% | 0.00% | 0.00% | 0.00% | 18.18% | 81.82% | Sufficient Agreement - May Pass; Review Comments | Revise |
|  | Lived Experience, Other | 0.00% | 0.00% | 0.00% | 0.00% | 0.00% | 100.00% |  |  |
|  | Service Delivery | 0.00% | 0.00% | 0.00% | 0.00% | 50.00% | 50.00% |  |  |
|  | Policy/Decision Maker | 0.00% | 0.00% | 0.00% | 10.00% | 0.00% | 90.00% |  |  |
|  | Researcher | 0.00% | 0.00% | 3.23% | 16.13% | 0.00% | 80.65% |  |  |
| **5.5** | Lived Experience, Self | 0.00% | 0.00% | 0.00% | 0.00% | 18.18% | 81.82% | Complete Agreement - Pass | Pass |
|  | Lived Experience, Other | 0.00% | 0.00% | 0.00% | 0.00% | 8.33% | 91.67% |  |  |
|  | Service Delivery | 0.00% | 0.00% | 0.00% | 0.00% | 0.00% | 100.00% |  |  |
|  | Policy/Decision Maker | 0.00% | 0.00% | 0.00% | 27.27% | 0.00% | 72.73% |  |  |
|  | Researcher | 0.00% | 3.23% | 0.00% | 6.45% | 19.35% | 70.97% |  |  |

**Supplemental Table 2: Round 2 Likert Responses by Clause Item and by Stakeholder Group**

| **Clause Item** | **Stakeholder Group** | **Responses** | | | | | | **Score Decision** | **Review**  **Decision** |
| --- | --- | --- | --- | --- | --- | --- | --- | --- | --- |
|  |  | **1** | **2** | **3** | **4** | **5** | **6** |  |  |
| **1.1** | Lived Experience, Self | 0.00% | 0.00% | 0.00% | 0.00% | 0.00% | 100.00% | Sufficient Agreement - May Pass; Review Comments | Pass |
|  | Lived Experience, Other | 0.00% | 0.00% | 0.00% | 0.00% | 0.00% | 100.00% |  |  |
|  | Service Delivery | 0.00% | 0.00% | 0.00% | 0.00% | 21.87% | 78.13% |  |  |
|  | Policy/Decision Maker | 0.00% | 0.00% | 0.00% | 0.00% | 0.00% | 100.00% |  |  |
|  | Researcher | 0.00% | 0.00% | 0.00% | 0.00% | 41.67% | 58.33% |  |  |
|  | *Unknown | 0.00% | 0.00% | 0.00% | 0.00% | 0.00% | 100.00% |  |  |
| **1.2** | Lived Experience, Self | 0.00% | 0.00% | 0.00% | 0.00% | 16.67% | 83.33% | Sufficient Agreement - May Pass; Review Comments | Pass |
|  | Lived Experience, Other | 0.00% | 0.00% | 0.00% | 0.00% | 8.33% | 91.67% |  |  |
|  | Service Delivery | 0.00% | 0.00% | 0.00% | 3.13% | 18.75% | 78.13% |  |  |
|  | Policy/Decision Maker | 0.00% | 0.00% | 0.00% | 0.00% | 0.00% | 100.00% |  |  |
|  | Researcher | 0.00% | 0.00% | 0.00% | 0.00% | 33.33% | 66.67% |  |  |
|  | *Unknown | 0.00% | 0.00% | 0.00% | 0.00% | 0.00% | 100.00% |  |  |
| **1.3** | Lived Experience, Self | 0.00% | 0.00% | 0.00% | 9.09% | 27.27% | 63.64% | Sufficient Agreement - May Pass; Review Comments | Revise |
|  | Lived Experience, Other | 0.00% | 0.00% | 0.00% | 8.33% | 16.67% | 75.00% |  |  |
|  | Service Delivery | 3.12% | 3.12% | 6.25% | 0.00% | 21.87% | 65.62% |  |  |
|  | Policy/Decision Maker | 0.00% | 0.00% | 0.00% | 0.00% | 50.00% | 50.00% |  |  |
|  | Researcher | 0.00% | 0.00% | 8.33% | 0.00% | 58.33% | 33.33% |  |  |
|  | *Unknown | 0.00% | 0.00% | 0.00% | 0.00% | 100.00% | 0.00% |  |  |
| **1.4** | Lived Experience, Self | 0.00% | 0.00% | 0.00% | 0.00% | 9.09% | 90.91% | Complete Agreement - Pass | Pass |
|  | Lived Experience, Other | 0.00% | 0.00% | 0.00% | 0.00% | 8.33% | 91.67% |  |  |
|  | Service Delivery | 0.00% | 3.12% | 0.00% | 0.00% | 25.00% | 71.87% |  |  |
|  | Policy/Decision Maker | 0.00% | 0.00% | 0.00% | 0.00% | 0.00% | 100.00% |  |  |
|  | Researcher | 0.00% | 0.00% | 8.33% | 0.00% | 16.67% | 75.00% |  |  |
|  | *Unknown | 0.00% | 0.00% | 0.00% | 0.00% | 0.00% | 100.00% |  |  |
| **1.5** | Lived Experience, Self | 0.00% | 0.00% | 0.00% | 0.00% | 9.09% | 90.91% | Sufficient Agreement - May Pass; Review Comments | Revise |
|  | Lived Experience, Other | 0.00% | 0.00% | 0.00% | 8.33% | 8.33% | 83.33% |  |  |
|  | Service Delivery | 0.00% | 0.00% | 0.00% | 3.12% | 12.50% | 84.37% |  |  |
|  | Policy/Decision Maker | 0.00% | 0.00% | 0.00% | 0.00% | 50.00% | 50.00% |  |  |
|  | Researcher | 0.00% | 0.00% | 0.00% | 8.33% | 50.00% | 41.67% |  |  |
|  | *Unknown | 0.00% | 0.00% | 0.00% | 0.00% | 100.00% | 0.00% |  |  |
| **1.6** | Lived Experience, Self | 0.00% | 0.00% | 0.00% | 0.00% | 36.36% | 63.64% | Sufficient Agreement - May Pass; Review Comments | Pass |
|  | Lived Experience, Other | 0.00% | 0.00% | 0.00% | 0.00% | 8.33% | 91.67% |  |  |
|  | Service Delivery | 0.00% | 0.00% | 0.00% | 0.00% | 25.00% | 75.00% |  |  |
|  | Policy/Decision Maker | 0.00% | 0.00% | 0.00% | 0.00% | 0.00% | 100.00% |  |  |
|  | Researcher | 0.00% | 8.33% | 8.33% | 0.00% | 16.67% | 66.67% |  |  |
|  | *Unknown | 0.00% | 0.00% | 0.00% | 0.00% | 0.00% | 100.00% |  |  |
| **2.1** | Lived Experience, Self | 0.00% | 0.00% | 0.00% | 0.00% | 27.27% | 72.73% | Sufficient Agreement - May Pass; Review Comments | Revise |
|  | Lived Experience, Other | 0.00% | 0.00% | 0.00% | 0.00% | 16.67% | 83.33% |  |  |
|  | Service Delivery | 0.00% | 0.00% | 3.12% | 0.00% | 21.88% | 75.00% |  |  |
|  | Policy/Decision Maker | 0.00% | 0.00% | 0.00% | 0.00% | 0.00% | 100.00% |  |  |
|  | Researcher | 0.00% | 0.00% | 0.00% | 18.18% | 54.55% | 27.27% |  |  |
|  | *Unknown | 0.00% | 0.00% | 0.00% | 0.00% | 0.00% | 100.00% |  |  |
| **2.2 passed in Round 1** | | | | | | | | | |
| **2.3** | Lived Experience, Self | 0.00% | 0.00% | 0.00% | 0.00% | 18.18% | 81.82% | Sufficient Agreement - May Pass; Review Comments | Pass |
|  | Lived Experience, Other | 0.00% | 8.33% | 8.33% | 8.33% | 0.00% | 75.00% |  |  |
|  | Service Delivery | 0.00% | 0.00% | 0.00% | 0.00% | 31.25% | 68.75% |  |  |
|  | Policy/Decision Maker | 0.00% | 0.00% | 0.00% | 0.00% | 50.00% | 50.00% |  |  |
|  | Researcher | 0.00% | 0.00% | 0.00% | 0.00% | 27.27% | 72.73% |  |  |
|  | *Unknown | 0.00% | 0.00% | 0.00% | 0.00% | 0.00% | 100.00% |  |  |
| **2.4** | Lived Experience, Self | 0.00% | 0.00% | 0.00% | 0.00% | 9.09% | 90.91% | Complete Agreement - Pass | Pass |
|  | Lived Experience, Other | 0.00% | 0.00% | 0.00% | 0.00% | 8.33% | 91.67% |  |  |
|  | Service Delivery | 0.00% | 0.00% | 0.00% | 0.00% | 12.50% | 87.50% |  |  |
|  | Policy/Decision Maker | 0.00% | 0.00% | 0.00% | 0.00% | 0.00% | 100.00% |  |  |
|  | Researcher | 0.00% | 9.09% | 0.00% | 9.09% | 0.00% | 81.82% |  |  |
|  | *Unknown | 0.00% | 0.00% | 0.00% | 0.00% | 0.00% | 100.00% |  |  |
| **2.5 removed in revision (incorporated into other clause item)** | | | | | | | | | |
| **3.1** | Lived Experience, Self | 0.00% | 0.00% | 0.00% | 0.00% | 27.27% | 72.73% | Sufficient Agreement - May Pass; Review Comments | Pass |
|  | Lived Experience, Other | 0.00% | 0.00% | 0.00% | 0.00% | 8.33% | 91.67% |  |  |
|  | Service Delivery | 0.00% | 0.00% | 0.00% | 6.25% | 21.88% | 71.87% |  |  |
|  | Policy/Decision Maker | 0.00% | 0.00% | 0.00% | 0.00% | 50.00% | 50.00% |  |  |
|  | Researcher | 0.00% | 0.00% | 0.00% | 0.00% | 27.27% | 72.73% |  |  |
|  | *Unknown | 0.00% | 0.00% | 0.00% | 0.00% | 0.00% | 100.00% |  |  |
| **3.2** | Lived Experience, Self | 0.00% | 0.00% | 0.00% | 0.00% | 27.27% | 72.73% | Sufficient Agreement - May Pass; Review Comments | Revise |
|  | Lived Experience, Other | 0.00% | 0.00% | 0.00% | 0.00% | 0.00% | 100.00% |  |  |
|  | Service Delivery | 0.00% | 0.00% | 0.00% | 0.00% | 31.25% | 68.75% |  |  |
|  | Policy/Decision Maker | 0.00% | 0.00% | 0.00% | 0.00% | 50.00% | 50.00% |  |  |
|  | Researcher | 0.00% | 0.00% | 0.00% | 27.27% | 9.09% | 63.64% |  |  |
|  | *Unknown | 0.00% | 0.00% | 0.00% | 0.00% | 0.00% | 100.00% |  |  |
| **3.3 passed in Round 1** | | | | | | | | | |
| **4.1** | Lived Experience, Self | 0.00% | 0.00% | 9.09% | 9.09% | 0.00% | 81.82% | Sufficient Agreement - May Pass; Review Comments | Revise |
|  | Lived Experience, Other | 0.00% | 0.00% | 0.00% | 8.33% | 16.67% | 75.00% |  |  |
|  | Service Delivery | 0.00% | 0.00% | 0.00% | 6.25% | 15.62% | 78.12% |  |  |
|  | Policy/Decision Maker | 0.00% | 0.00% | 0.00% | 0.00% | 50.00% | 50.00% |  |  |
|  | Researcher | 0.00% | 0.00% | 9.09% | 0.00% | 9.09% | 81.82% |  |  |
|  | *Unknown | 0.00% | 0.00% | 0.00% | 0.00% | 0.00% | 100.00% |  |  |
| **4.2** | Lived Experience, Self | 0.00% | 0.00% | 0.00% | 0.00% | 9.09% | 90.91% | Insufficient Agreement - 1+ Group Disagrees - Revise | Revise |
|  | Lived Experience, Other | 0.00% | 0.00% | 0.00% | 0.00% | 0.00% | 100.00% |  |  |
|  | Service Delivery | 0.00% | 0.00% | 0.00% | 0.00% | 9.37% | 90.62% |  |  |
|  | Policy/Decision Maker | 0.00% | 0.00% | 0.00% | 50.00% | 0.00% | 50.00% |  |  |
|  | Researcher | 0.00% | 18.18% | 9.09% | 0.00% | 27.27% | 45.45% |  |  |
|  | *Unknown | 0.00% | 0.00% | 0.00% | 0.00% | 0.00% | 100.00% |  |  |
| **4.3** | Lived Experience, Self | 0.00% | 0.00% | 0.00% | 9.09% | 27.27% | 63.64% | Sufficient Agreement - May Pass; Review Comments | Revise |
|  | Lived Experience, Other | 0.00% | 0.00% | 8.33% | 0.00% | 0.00% | 91.67% |  |  |
|  | Service Delivery | 0.00% | 0.00% | 0.00% | 6.25% | 18.75% | 75.00% |  |  |
|  | Policy/Decision Maker | 0.00% | 0.00% | 0.00% | 0.00% | 0.00% | 100.00% |  |  |
|  | Researcher | 0.00% | 0.00% | 0.00% | 18.18% | 18.18% | 63.64% |  |  |
|  | *Unknown | 0.00% | 0.00% | 0.00% | 0.00% | 0.00% | 100.00% |  |  |
| **4.4 removed in revision (incorporated into other clause item)** | | | | | | | | | |
| **4.5** | Lived Experience, Self | 0.00% | 0.00% | 0.00% | 0.00% | 0.00% | 100.00% | Sufficient Agreement - May Pass; Review Comments | Pass |
|  | Lived Experience, Other | 0.00% | 0.00% | 0.00% | 0.00% | 0.00% | 100.00% |  |  |
|  | Service Delivery | 0.00% | 0.00% | 0.00% | 3.12% | 15.63% | 81.25% |  |  |
|  | Policy/Decision Maker | 0.00% | 0.00% | 0.00% | 0.00% | 0.00% | 100.00% |  |  |
|  | Researcher | 0.00% | 0.00% | 0.00% | 9.09% | 45.45% | 45.45% |  |  |
|  | *Unknown | 0.00% | 0.00% | 0.00% | 0.00% | 0.00% | 100.00% |  |  |
| **4.6 removed in revision (incorporated into other clause item)** | | | | | | | | | |
| **4.7 removed in revision (incorporated into other clause item)** | | | | | | | | | |
| **4.8 passed in Round 1** | | | | | | | | | |
| **4.9** | Lived Experience, Self | 0.00% | 0.00% | 0.00% | 9.09% | 9.09% | 81.82% | Complete Agreement - Pass | Pass |
|  | Lived Experience, Other | 0.00% | 0.00% | 0.00% | 0.00% | 8.33% | 91.67% |  |  |
|  | Service Delivery | 0.00% | 0.00% | 0.00% | 0.00% | 12.50% | 87.50% |  |  |
|  | Policy/Decision Maker | 0.00% | 0.00% | 0.00% | 0.00% | 0.00% | 100.00% |  |  |
|  | Researcher | 0.00% | 0.00% | 0.00% | 9.09% | 0.00% | 90.91% |  |  |
|  | *Unknown | 0.00% | 0.00% | 0.00% | 0.00% | 0.00% | 100.00% |  |  |
| **4.10 removed in revision (incorporated into other clause item)** | | | | | | | | | |
| **5.1** | Lived Experience, Self | 0.00% | 0.00% | 0.00% | 0.00% | 18.18% | 81.82% | Complete Agreement - Pass | Pass |
|  | Lived Experience, Other | 0.00% | 0.00% | 0.00% | 0.00% | 0.00% | 100.00% |  |  |
|  | Service Delivery | 0.00% | 0.00% | 0.00% | 0.00% | 6.25% | 93.75% |  |  |
|  | Policy/Decision Maker | 0.00% | 0.00% | 0.00% | 0.00% | 0.00% | 100.00% |  |  |
|  | Researcher | 0.00% | 0.00% | 0.00% | 9.09% | 18.18% | 72.73% |  |  |
|  | *Unknown | 0.00% | 0.00% | 0.00% | 0.00% | 0.00% | 100.00% |  |  |
| **5.2** | Lived Experience, Self | 0.00% | 0.00% | 0.00% | 0.00% | 9.09% | 90.91% | Complete Agreement - Pass | Pass |
|  | Lived Experience, Other | 0.00% | 0.00% | 0.00% | 0.00% | 16.67% | 83.33% |  |  |
|  | Service Delivery | 0.00% | 0.00% | 0.00% | 3.12% | 6.25% | 90.63% |  |  |
|  | Policy/Decision Maker | 0.00% | 0.00% | 0.00% | 0.00% | 0.00% | 100.00% |  |  |
|  | Researcher | 0.00% | 0.00% | 0.00% | 0.00% | 27.27% | 72.73% |  |  |
|  | *Unknown | 0.00% | 0.00% | 0.00% | 0.00% | 0.00% | 100.00% |  |  |
| **5.3** | Lived Experience, Self | 0.00% | 0.00% | 0.00% | 0.00% | 9.09% | 90.91% | Complete Agreement - Pass | Pass |
|  | Lived Experience, Other | 0.00% | 0.00% | 0.00% | 0.00% | 0.00% | 100.00% |  |  |
|  | Service Delivery | 0.00% | 0.00% | 0.00% | 0.00% | 18.75% | 81.25% |  |  |
|  | Policy/Decision Maker | 0.00% | 0.00% | 0.00% | 0.00% | 0.00% | 100.00% |  |  |
|  | Researcher | 0.00% | 0.00% | 0.00% | 0.00% | 18.18% | 81.82% |  |  |
|  | *Unknown | 0.00% | 0.00% | 0.00% | 0.00% | 0.00% | 100.00% |  |  |
| **5.4** | Lived Experience, Self | 0.00% | 0.00% | 0.00% | 0.00% | 9.09% | 90.91% | Complete Agreement - Pass | Pass |
|  | Lived Experience, Other | 0.00% | 0.00% | 0.00% | 0.00% | 0.00% | 100.00% |  |  |
|  | Service Delivery | 0.00% | 0.00% | 0.00% | 6.25% | 0.00% | 93.75% |  |  |
|  | Policy/Decision Maker | 0.00% | 0.00% | 0.00% | 0.00% | 0.00% | 100.00% |  |  |
|  | Researcher | 0.00% | 0.00% | 0.00% | 0.00% | 0.00% | 100.00% |  |  |
|  | *Unknown | 0.00% | 0.00% | 0.00% | 0.00% | 0.00% | 100.00% |  |  |
| **5.5 passed in Round 1** | | | | | | | | | |

**Supplemental Table 3: Round 3 Likert Responses by Clause Item and by Stakeholder Group**

| **Clause Item** | **Stakeholder Group** | **Responses** | | | | | | **Score**  **Decision** | **Review**  **Decision** |
| --- | --- | --- | --- | --- | --- | --- | --- | --- | --- |
|  |  | **1** | **2** | **3** | **4** | **5** | **6** |  |  |
| **1.1 passed in Round 2** | | | | | | | | | |
| **1.2 passed in Round 2** | | | | | | | | | |
| **1.3** | Lived Experience, Self | 0.00% | 0.00% | 0.00% | 0.00% | 18.18% | 81.82% | Sufficient Agreement - May Pass; Review Comments | Pass |
|  | Lived Experience, Other | 0.00% | 0.00% | 0.00% | 0.00% | 0.00% | 100.00% |  |  |
|  | Service Delivery | 3.12% | 0.00% | 0.00% | 0.00% | 15.62% | 81.25% |  |  |
|  | Policy/Decision Maker | 0.00% | 0.00% | 0.00% | 0.00% | 50.00% | 50.00% |  |  |
|  | Researcher | 0.00% | 0.00% | 0.00% | 0.00% | 25.00% | 75.00% |  |  |
|  | *Unknown | 0.00% | 0.00% | 0.00% | 0.00% | 100.00% | 0.00% |  |  |
| **1.4 passed in Round 2** | | | | | | | | | |
| **1.5** | Lived Experience, Self | 0.00% | 0.00% | 9.09% | 0.00% | 9.09% | 81.82% | Sufficient Agreement - May Pass; Review Comments | Pass |
|  | Lived Experience, Other | 0.00% | 0.00% | 0.00% | 0.00% | 0.00% | 100.00% |  |  |
|  | Service Delivery | 0.00% | 0.00% | 0.00% | 0.00% | 12.50% | 87.50% |  |  |
|  | Policy/Decision Maker | 0.00% | 0.00% | 0.00% | 0.00% | 50.00% | 50.00% |  |  |
|  | Researcher | 0.00% | 0.00% | 8.33% | 8.33% | 25.00% | 58.33% |  |  |
|  | *Unknown | 0.00% | 0.00% | 0.00% | 0.00% | 100.00% | 0.00% |  |  |
| **1.6 passed in Round 2** | | | | | | | | | |
| **2.1** | Lived Experience, Self | 0.00% | 0.00% | 0.00% | 0.00% | 18.18% | 81.82% | Sufficient Agreement - May Pass; Review Comments | Pass |
|  | Lived Experience, Other | 0.00% | 0.00% | 0.00% | 0.00% | 8.33% | 91.67% |  |  |
|  | Service Delivery | 0.00% | 0.00% | 0.00% | 0.00% | 6.25% | 93.75% |  |  |
|  | Policy/Decision Maker | 0.00% | 0.00% | 0.00% | 0.00% | 0.00% | 100.00% |  |  |
|  | Researcher | 0.00% | 0.00% | 0.00% | 9.09% | 36.36% | 54.55% |  |  |
|  | *Unknown | 0.00% | 0.00% | 0.00% | 0.00% | 0.00% | 100.00% |  |  |
| **2.2 passed in Round 1** | | | | | | | | | |
| **2.3 passed in Round 2** | | | | | | | | | |
| **2.4 passed in Round 2** | | | | | | | | | |
| **2.5 removed in revision (incorporated into other clause item)** | | | | | | | | | |
| **3.1 passed in Round 2** | | | | | | | | | |
| **3.2** | Lived Experience, Self | 0.00% | 0.00% | 0.00% | 0.00% | 18.18% | 81.82% | Sufficient Agreement - May Pass; Review Comments | Pass |
|  | Lived Experience, Other | 0.00% | 0.00% | 0.00% | 0.00% | 0.00% | 100.00% |  |  |
|  | Service Delivery | 0.00% | 0.00% | 0.00% | 0.00% | 18.75% | 81.25% |  |  |
|  | Policy/Decision Maker | 0.00% | 0.00% | 0.00% | 0.00% | 50.00% | 50.00% |  |  |
|  | Researcher | 0.00% | 0.00% | 0.00% | 18.18% | 18.18% | 63.64% |  |  |
|  | *Unknown | 0.00% | 0.00% | 0.00% | 0.00% | 0.00% | 100.00% |  |  |
| **3.3 passed in Round 1** | | | | | | | | | |
| **4.1** | Lived Experience, Self | 0.00% | 0.00% | 9.09% | 0.00% | 0.00% | 90.91% | Sufficient Agreement - May Pass; Review Comments | Pass |
|  | Lived Experience, Other | 0.00% | 0.00% | 8.33% | 0.00% | 8.33% | 83.33% |  |  |
|  | Service Delivery | 0.00% | 0.00% | 0.00% | 3.12% | 15.63% | 81.25% |  |  |
|  | Policy/Decision Maker | 0.00% | 0.00% | 0.00% | 0.00% | 50.00% | 50.00% |  |  |
|  | Researcher | 0.00% | 0.00% | 9.09% | 0.00% | 0.00% | 90.91% |  |  |
|  | *Unknown | 0.00% | 0.00% | 0.00% | 0.00% | 0.00% | 100.00% |  |  |
| **4.2** | Lived Experience, Self | 0.00% | 0.00% | 0.00% | 0.00% | 9.09% | 90.91% | Insufficient Agreement - 1+ Group Disagrees - Revise | Pass |
|  | Lived Experience, Other | 0.00% | 0.00% | 0.00% | 0.00% | 0.00% | 100.00% |  |  |
|  | Service Delivery | 0.00% | 0.00% | 0.00% | 0.00% | 6.25% | 93.75% |  |  |
|  | Policy/Decision Maker | 0.00% | 0.00% | 0.00% | 50.00% | 0.00% | 50.00% |  |  |
|  | Researcher | 0.00% | 18.18% | 9.09% | 0.00% | 9.09% | 63.64% |  |  |
|  | *Unknown | 0.00% | 0.00% | 0.00% | 0.00% | 0.00% | 100.00% |  |  |
| **4.3** | Lived Experience, Self | 0.00% | 9.09% | 0.00% | 0.00% | 18.18% | 72.73% | Complete Agreement - Pass | Pass |
|  | Lived Experience, Other | 0.00% | 0.00% | 0.00% | 8.33% | 0.00% | 91.67% |  |  |
|  | Service Delivery | 0.00% | 3.12% | 0.00% | 3.12% | 18.75% | 75.00% |  |  |
|  | Policy/Decision Maker | 0.00% | 0.00% | 0.00% | 0.00% | 0.00% | 100.00% |  |  |
|  | Researcher | 0.00% | 0.00% | 0.00% | 9.09% | 18.18% | 72.73% |  |  |
|  | *Unknown | 0.00% | 0.00% | 0.00% | 0.00% | 0.00% | 100.00% |  |  |
| **4.4 removed in revision (incorporated into other clause item)** | | | | | | | | | |
| **4.5 passed in Round 2** | | | | | | | | | |
| **4.6 removed in revision (incorporated into other clause item)** | | | | | | | | | |
| **4.7 removed in revision (incorporated into other clause item)** | | | | | | | | | |
| **4.8 passed in Round 1** | | | | | | | | | |
| **4.9 passed in Round 2** | | | | | | | | | |
| **4.10 removed in revision (incorporated into other clause item)** | | | | | | | | | |
| **5.1 passed in Round 2** | | | | | | | | | |
| **5.2 passed in Round 2** | | | | | | | | | |
| **5.3 passed in Round 2** | | | | | | | | | |
| **5.4 passed in Round 2** | | | | | | | | | |
| **5.5 passed in Round 1** | | | | | | | | | |
